# Decalogue of Catatonia in Autism Spectrum Disorders

**DOI:** 10.3389/fpsyt.2014.00157

**Published:** 2014-11-06

**Authors:** Dirk M. Dhossche

**Affiliations:** ^1^University of Mississippi Medical Center, Jackson, MS, USA

**Keywords:** catatonia, autism spectrum disorders, treatment, benzodiazepines, electroconvulsive therapy

Plaisante justice, qu’une rivière borne!Vérité au-deçà des Pyrénées, erreur au-delà.

Funny justice that is marked by a river!What is truth on this side of the Pyrenees is a mistake on the other side. (Translation by DMD)

From the book “Pensées” by Blaise Pascal (1623–1662)

French mathematician, physicist, inventor, writer, and philosopher.

This article is an unabashed drumroll for increased recognition and treatment of catatonia in autism spectrum disorders (ASD). This new diagnostic and treatment paradigm has emerged during the last decade (1, 2) and is supported by changes in catatonia classification in DSM-5 (3) purporting to boost recognition of pediatric catatonia and catatonia in ASD (4). Findings are summarized, a vignette is presented, and a Decalogue (or “10 commandments”) is offered covering rules for assessment, diagnosis, treatment, and research in the field of catatonia in ASD. Unlike the biblical Decalogue handed to Moses on Mount Sinai, these rules are hardly divine, do not mark universal truths, are mainly based on clinical experience, and need to be calibrated further as knowledge increases. They do represent the best available recommendations, at least in my opinion, for future research and in order to achieve positive outcomes that are equally rewarding for the patients and their families, and for their physicians.

## Prevalence, Assessment, and Treatment of Catatonia in ASD

Catatonia is a severe but treatable syndrome that warrants prompt diagnosis and treatment with benzodiazepines and electroconvulsive therapy (ECT) and that occurs in patients of all ages, including children and adolescents (5–7). The syndrome becomes critical and life threatening in its malignant form when aggravated by fever and autonomic dysfunction. Advances in catatonia research have segued into the field of autism over the last 10 years. Catatonia in ASD has been increasingly recognized at a rate of 4–17% in adolescents and adults with ASD (Table 1). No systematic studies have been done in preadolescent and older (>60 years old) patients. The vignette of a 10-year-old boy with catatonia and ASD that is presented below shows its occurrence in preadolescence. There are no cases in the literature, to my knowledge, describing catatonia in patients with ASD older than 60.

**Table 1:** **Prevalence studies of catatonia in autism spectrum disorders**.

Reference	*N*	Design sample population (age, range, and/or mean)	% With catatonia (age, range, and/or mean)
Wing and Shah (8)	506	Cross-sectional referred sample with autism (NA)	17 (Age range 15–50 years, mean age = 24.6)
Billstedt et al. (9)	120	Longitudinal population sample with autism (age range 17–40 years, mean age = 25.5)	12 (NA)
Ohta et al. (10)	69	Cross-sectional referred sample with autism (NA)	12 (Age range 21–40 years, mean age = 28 SD = 5.5)
Hutton et al. (11)	135	Longitudinal referred sample with autism (age range 21–57 years, mean age = 35)	4 (NA)
Ghaziuddin et al. (12)	81	Cross-sectional referred sample with autism (mean age = 14, SD = 2)	16 (NA)

*NA, not available*.

Symptoms that should alert the clinician for catatonia in adolescents and young adults with ASD are markedly increased psychomotor slowness, which may alternate with excessive motor activity, apparently purposeless, and not influenced by external stimuli, extreme negativism or muteness, stereotypy, peculiarities of voluntary movement, echolalia, or echopraxia. The diagnosis of catatonia in ASD is made applying known clinical signs of catatonia and using standardized catatonia rating scales to assess the scope and the severity of the symptoms. In addition, the diagnosis may be supported by a benzodiazepine (most commonly lorazepam) challenge test, which is known to result in a marked, albeit temporary, improvement.

Stressful life events, the loss of routine, experiences of loss, interpersonal conflicts, and discrepancies between the ability in the patient and parental expectations, especially in higher functioning autistic youth, may precipitate catatonia, which may present in conjunction with other major psychiatric or medical disorders (13, 14). A medical work-up and comprehensive drug screening is necessary to uncover medical or toxic conditions.

Benzodiazepines and bilateral ECT, including maintenance ECT, should be considered as safe and effective medical treatments for pediatric catatonia and catatonia in ASD based on case-reports and case-series (2, 15). Milder cases have improved with psychological–behavioral interventions (16). Benzodiazepines, most commonly lorazepam, are the first-line treatment, followed by ECT if benzodiazepines are not or insufficiently effective. The duration and intensity of ECT varies in each case. Although there are reports of a small number of cases that show the effective use of right unilateral ECT in catatonia (17), none of these patients were diagnosed with ASD. In almost all cases with ASD, bilateral ECT has been used (18) and should be considered as the preferred method until further studies or experience shows advantages when using or starting with unilateral ECT. So far concerns of cognitive impairment with bilateral ECT have not emerged. There are now a few reported cases that have continued to receive bilateral ECT for several years to maintain improvement and to prevent relapse without any indication of cognitive or neuropsychological worsening (19, 20).

DeJong et al. (18) have recently reviewed the literature on the efficacy of various treatments for catatonia in ASD. They found 22 pertinent articles describing the treatment of 28 pediatric and adult patients supporting the use of ECT, high-dose lorazepam, and behavioral therapy. They deplore the scarcity of papers and in-depth analyses. They conclude that controlled multi-centers studies are warranted to compare treatments and to determine the optimal treatment for catatonia in ASD.

## Vignette

This vignette of a 10-year-old boy diagnosed at age 4 with high-functioning ASD, type “Asperger,” who developed full-blown catatonia over the course of a few months, is the synopsis of a case-report that is in press (21).

At 4 years of age, A was diagnosed with ASD due to restrictive interests, vocal and motor tics, specific phobias, attention deficits, severe aggression, obsessive–compulsive tendencies, and abnormal movements in mouth, and repetitive movements. Testing showed an IQ of 80. At the age of 10, 1 year prior to admission for catatonia, A became extremely upset about an incident on school when his best friend spit on his thermos. Over the next few days, he became increasingly and excessively fearful that people were deliberately spitting on him. He stated that the spit could find its way into his mouth and then brain where it would ruin his identity. He refused to leave his room and go to school and went into violent rages. Over the next 6 months, several interventions and medications, including SSRI’s and antipsychotics, were tried to no avail. A medical work-up included blood work, comprehensive drug testing, brain imaging and lumbar puncture, autoimmune antibodies [lupus serology, pediatric autoimmune neuropsychiatric disorders associated with streptococcal infections (PANDAS) serology, anti-*N*-methyl-d-aspartate (NMDA) receptor antibodies] and was completely negative.

Six months after onset of symptoms, A started to have episodes lasting several days where he would stop speaking, refusing to eat, and sitting in the same spot in the hallway. He was diagnosed with catatonia and was started on lorazepam with positive effects but the patient took the medication inconsistently. His condition worsened. A trial of high-dose-lorazepam given intramuscularly was done, with maximum dose of 24 mg (8 mg IM three times per day), with only partial response yet no sedation. Ten months after onset of symptoms, ECT was started with consent of the parents. Bilateral ECT was started three times per week. After three ECT treatments, A started to speak a few words occasionally, mostly echoing the words (and body movements) of others. After six treatments, he started to allow others to touch him and was spitting less but continued to need help in all areas of daily function. Over the next 2 weeks ECT was continued and aripiprazole was started as an antipsychotic adjunct and titrated to 20 mg/day.

A continued to make slow progress until he became fully verbal on day 31 of admission after the 12th ECT. He carried out a long conversation with his teacher. He knew that he was in the hospital, and was oriented to season but not the month or day. A stated that he was still concerned that he would die if someone spit on him. During the next week, he improved rapidly and returned to premorbid functioning. He was discharged after two more ECT treatments. Throughout the course of ECT, no side effects were noted except an occasional headache in the afternoon on the day of ECT. His memory and cognitive function seemed greatly improved over the course of admission and ECT. At 2-year follow-up, A has had no relapses of catatonia. Maintenance therapy consists of lorazepam (4 mg/day) and aripiprazole (15 mg/day). A has been able to resume school in special education classes. The parents report that he has been back to baseline since more than 1 year.

## Decalogue of Catatonia in Autism Spectrum Disorders

1. Catatonia is a diagnosable syndrome in ASD (2).
2. Patients with ASD are prone to develop catatonia due to concurrent medical and psychological impairments (13, 22).
3. The treatment of catatonia in ASD is very specific and consists of benzodiazepines and ECT (23).
4. Some severe forms of repetitive self-injurious behavior (SIB) are best conceptualized as catatonic stereotypy (with bodily injury) for which benzodiazepines, ECT, and maintenance ECT are indicated (24, 25).
5. Do not use antipsychotics in the active phase of catatonia before benzodiazepines or ECT is started, in order to avoid malignant catatonia and neuroleptic malignant syndrome (23).
6. A lorazepam (initial administration of 1 or 2 mg po or parentally followed by increased doses up to 20–30 mg/day depending on the level of response and side effects or sedation) or zolpidem (5 or 10 mg po) challenge test verifies the catatonia diagnosis (23).
7. High dosages of lorazepam, up to 20–30 mg daily, may be necessary for full symptom resolution (23) and, surprisingly, are well tolerated without sedation by some patients.
8. Bilateral ECT is the definitive treatment for catatonia in ASD when lorazepam does not bring about swift or sufficient relief or when fever and autonomic dysfunction arise. Concurrent and synergistic use of lorazepam and ECT is possible when using flumazenil to temporarily suspend the anticonvulsant effects of benzodiazepines during ECT (23).
9. Maintenance ECT is a safe treatment option that is sometimes crucial to avoid relapse (19, 20).
10. Catatonia provides a window into the mechanism of autism, and vice versa (26, 27).

## Rosetta Stone

Catatonia is characterized by repetitive movements, mutism, posturing, and frantic agitation. These signs are also frequent in autism yet usually do not amount to a diagnosis of catatonia unless there is a sharp and sustained increase of these symptoms lasting days or weeks. Catatonia and autism have widely different historical roots (28). Much can be learned from the study of catatonia in ASD. The Rosetta stone was a small stone tablet containing the same message written in Greek and two different Egyptian scripts. The stone was essential for deciphering Egyptian hieroglyphics. Can the group of patients who are diagnosed with both autism and catatonia be like the Rosetta stone, providing the complete sentences required for deciphering the mechanisms of autism and catatonia? Do the syndromes have a common pathophysiology? Can the successful treatment of catatonia be applied to all patients that meet criteria for autism and catatonia? Can early application of treatments for catatonia stop autistic regression? These questions beg answers sorely needed by patients and their families.

Blaise Pascal lamented in the seventeenth century over the vagaries of justice. He observed that blasphemy in one place (or time) is truth in another place (or time). The situation is not different in medicine and also seems to apply to catatonia in ASD. Detractors interpret catatonia as an aspecific and undefined epiphenomenon of another “more valid” disorder such as a medical disorder, schizophrenia, bipolar disorder, or obsessive–compulsive disorder, requiring no specific treatment. For example, in the follow-up study of Hutton et al. (11) of 135 individuals with ASD to at least the age of 21 years, five individuals had a relatively sudden onset of catatonia and obsessive–compulsive disorder. In two cases, the disorders resolved with treatment but in the other three there was only a partial recovery. None of these patients was treated with benzodiazepines or ECT. The authors ask themselves whether benzodiazepines or ECT should have been used in these cases but then demur: “*If the impression that catatonia develops as a result of obsessive-compulsive phenomena is correct, this would not seem a strong indication. It should be added that catatonia lacks a clear definition and there would be a danger of moving too readily to heroic interventions of unproven value*.” Instead, they recommend the pharmacological and psychological approaches for obsessive–compulsive disorders unassociated with autism (or catatonia). However, the vignette and similar cases support the use of ECT in cases of ASD with catatonia, also with concurrent onset or increase of obsessions and compulsions. Of course, unfounded ethical objections to pediatric ECT, legal restrictions on pediatric ECT, lack of access to ECT in general, and stigma remain salient obstacles to effective treatment of catatonia in these challenging patients (29, 30).

## Conflict of Interest Statement

The author declares that the research was conducted in the absence of any commercial or financial relationships that could be construed as a potential conflict of interest.
